# Predicting drug–protein interactions by preserving the graph information of multi source data

**DOI:** 10.1186/s12859-023-05620-6

**Published:** 2024-01-04

**Authors:** Jiahao Wei, Linzhang Lu, Tie Shen

**Affiliations:** 1 School of Mathematical Sciences, Guizhou Normal University, Guiyang, 550025 China; 2 School of Mathematical Sciences, Xiamen University, Xiamen, 361005 China; 3 Key Laboratory of Information and Computing Science Guizhou Province, Guizhou Normal University, Guizhou, 550001 China

**Keywords:** Drug–target interactions, Graph attention networks, Residual graph convolutional neural networks

## Abstract

Examining potential drug–target interactions (DTIs) is a pivotal component of drug discovery and repurposing. Recently, there has been a significant rise in the use of computational techniques to predict DTIs. Nevertheless, previous investigations have predominantly concentrated on assessing either the connections between nodes or the consistency of the network’s topological structure in isolation. Such one-sided approaches could severely hinder the accuracy of DTI predictions. In this study, we propose a novel method called TTGCN, which combines heterogeneous graph convolutional neural networks (GCN) and graph attention networks (GAT) to address the task of DTI prediction. TTGCN employs a two-tiered feature learning strategy, utilizing GAT and residual GCN (R-GCN) to extract drug and target embeddings from the diverse network, respectively. These drug and target embeddings are then fused through a mean-pooling layer. Finally, we employ an inductive matrix completion technique to forecast DTIs while preserving the network’s node connectivity and topological structure. Our approach demonstrates superior performance in terms of area under the curve and area under the precision–recall curve in experimental comparisons, highlighting its significant advantages in predicting DTIs. Furthermore, case studies provide additional evidence of its ability to identify potential DTIs.

## Introduction

Predicting the existence of unknown drug–target interactions (DTIs) is a pivotal component of drug discovery and repurposing [1]. Identifying DTI has significant implications in drug repurposing [2] and drug discovery [3]. However, exploring the interactions between drugs and proteins with complex chemical properties is a challenging task [4]. Therefore, many studies use computer technology to design corresponding algorithms to solve biological problems [5] and predict unknown DTIs [6]. This enables biologists to acquire dependable drug–protein pairs, cutting down on the time and expenses required for DTI identification via biochemical experiments [7].

In the early stages of computational DTI prediction, two main types of methods were predominantly employed: docking simulations and ligand-based approaches [8, 9]. Docking techniques necessitate simulating the 3D structure of the target, yet not all target protein structures are available. Conversely, ligand-based approaches involve comparing the target protein of interest with a group of known target proteins for a specific ligand. However, ligand-based methods tend to provide less accurate predictions in situations where the number of known binding ligands is restricted.

Lately, there has been a growing inclination towards examining DTIs from a network-oriented standpoint [10]. This approach amalgamates diverse data from the heterogeneous drug–target network to evaluate the potential interaction probability for each drug–target pair [11]. For instance, Bleakley et al. introduced a support vector machine framework known as the bipartite local model (BLM) for DTI prediction [12]. However, this method involves large-scale high-order matrix computations, which often suffer from limited computational resources. Zheng et al. introduced a DTI identification model known as collaborative matrix factorization (CMF), which utilizes heterogeneous information networks for DTI prediction [13]. However, it does not consider the heterogeneity of information in each network, and fails to obtain effective feature representations of nodes. Olayan et al. developed DDR, an random forest-based ensemble learning algorithm that effectively mitigates the impact of class imbalance [14]. Nevertheless, the random forest’s straightforward voting mechanism places limitations on the performance of DDR. Furthermore, all these techniques are shallow models, which means they cannot fully delve into the intricate relationships between drugs and their respective targets.

Nevertheless, these methods predominantly depend on the similarity and interaction data of drug proteins, often overlooking the potential insights from other available data sources. In contrast, Luo et al. introduced a novel prediction methodology grounded in heterogeneous networks (DTINet) [15]. This method extensively leverages diverse relationships among drugs, proteins, and diseases. By acquiring low-dimensional vector representations of nodes, DTINet effectively predicts drug–protein interactions. The intricate associations inherent in drug and protein-related information pose a challenge for conventional methods, which frequently manifest as shallow prediction models struggling to grasp these intricate connections.

Hakime et al. presented DeepDTA, a deep learning model that relies solely on the sequence information of drugs and target proteins to forecast the binding affinity between them [16]. However, this method utilizes the molecular characteristics of drugs and proteins to predict DTI, while the information provided by similar molecules is ignored. Huang et al. introduced the molecular interaction transformer (MolTrans), which achieves more accurate and interpretable DTI prediction by capturing semantic relationships between substructures extracted from a large amount of unlabeled biomedical data [17]. Nonetheless, this method frequently neglects the three-dimensional spatial information of molecules, potentially constraining its efficacy in dealing with stereo-isomers and spatial interactions. During the same year, Sun et al. introduced an approach called autoencoder-based embedding fusion strategy (AEFS) for predicting DTIs. In this method, the initial drug characteristics are transformed into an embedding space using multiple encoders and then projected into a disease-related space through a decoder [18]. Nevertheless, it’s worth noting that these deep learning models overlook the topological characteristics of drugs and proteins, thus missing out on capturing their intricate interactions.

Sun et al. proposed GANDTI, a graph convolutional autoencoders and generative adversarial networks-based method for DTI prediction [19]. However, it fell short in harnessing information concerning disease associations linked to drugs and proteins, consequently failing to encapsulate the intricate relationships between drugs and proteins. Peng et al. introduced the Domain Interaction-based Heterogeneous Graph Convolutional Network (NIHGCN) for end-to-end prediction of anti-cancer drug response [20]. However, these methods only utilize GCN and overlook whether two nodes have consistent topological structures between them. During the same year, Li et al. introduced an innovative approach named IMCHGAN for DTI prediction. This method fine-tunes both the prediction scoring model and feature representation learning model using backpropagation to optimize their parameters [21]. Nevertheless, these methods predominantly center on evaluating the coherence of connections between nodes or isolated network topological structures. This unilateral approach could substantially impede the accuracy of DTI prediction.

**Fig. 1:**
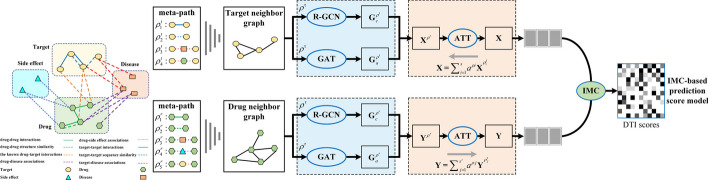
The framework of TTGCN

In order to address the constraints of existing DTI prediction techniques, we propose a new method in this study called TTGCN. As shown in Fig. 1, it combines heterogeneous graph convolutional neural networks and graph attention networks to address the DTI prediction problem. TTGCN utilizes graph attention networks (GAT) to preserve the connectivity between nodes and residual graph convolutional neural networks (R-GCN) to ensure the existence of connections between two nodes in both the original feature space and the embedding space. This guarantees the preservation of a consistent topological structure between the original feature space and the embedding space. Subsequently, we apply inductive matrix completion for DTI prediction. Our experimental comparisons indicate that our approach surpasses other methods in AUC and AUPR performance, underscoring its notable advantages in DTI prediction. Further validation through case studies confirms its effectiveness in identifying potential DTIs.

## Materials and method

Our primary goal is to forecast DTIs by analyzing the connections between proteins, diseases, drugs, and drug side effects. To accomplish this, we created a bio-heterogeneous network encompassing drugs and targets, extracting both edge information (network topology) and node information (node attributes) from this network. We harnessed the power of GAT and residual graph convolutional neural networks to generate embeddings for each node within the network. The interaction score is subsequently calculated based on the embeddings of both drugs and proteins.

### Dataset

The dataset utilized in this study was acquired from multiple scholarly articles. In their publication, Wishart et al. [22] introduced DrugBank as a comprehensive database that encompasses many details pertaining to medications, such as molecular structure and target proteins. The UniProt database, as proposed by the UniProt Consortium, serves as a comprehensive repository of protein-related data derived from scientific literature and experimental investigations [23]. In their study, O. Ursu et al. introduced DrugCentral [24], a comprehensive drug database that encompasses a wide range of information pertaining to pharmaceutical substances.

### The drug–target biological heterogeneous network

To integrate the specifics of various types of biological entities, a heterogeneous network is constructed. The drugs, proteins, diseases, and adverse results are regarded as the nodes in the network. The inter and intra relationships between these nodes are set as the edges in the network.

To enhance the extraction of network topology information, we introduce the concept of metapaths.

In heterogeneous information networks, metapaths refer to various semantic paths connecting two nodes [25]. A metapath is a composite relationship path between two nodes denoted as $\rho$. It is represented as $\rho =T_{1} \overset{sem1}{-}\ T_{2} \overset{sem2}{-}\ \cdots \overset{seml}{-}\ T_{l}$ (short for semantic), where $T_{i}$ represents different node types such as disease, drug, or target. These metapaths encapsulate unique semantic connections between various types of nodes. As illustrated in Fig. 1, the metapath $r \overset{inter}{-}\ r$ represents the direct interaction relationship between two drugs, such as $r_{2} \overset{inter}{-}\ r_{4}$. The metapath $r \overset{assoc}{-}\ d \overset{assoc}{-}r$ represents the relationship between two drugs and a common disease, such as $r_{3} \overset{assoc}{-}\ d_{1} \overset{assoc}{-}r_{4}$. It is evident that the semantic connections under these metapaths are not completely identical.

Next, we defined metapath-based neighbors as a set of nodes connected to a particular node through specific metapaths within the heterogeneous information network. Specifically, for a given node *i* and metapath $\rho$, denoted as $N^{p} (i)$, it represents the set of nodes connected to node *i* through metapath $\rho$.

**Fig. 2:**
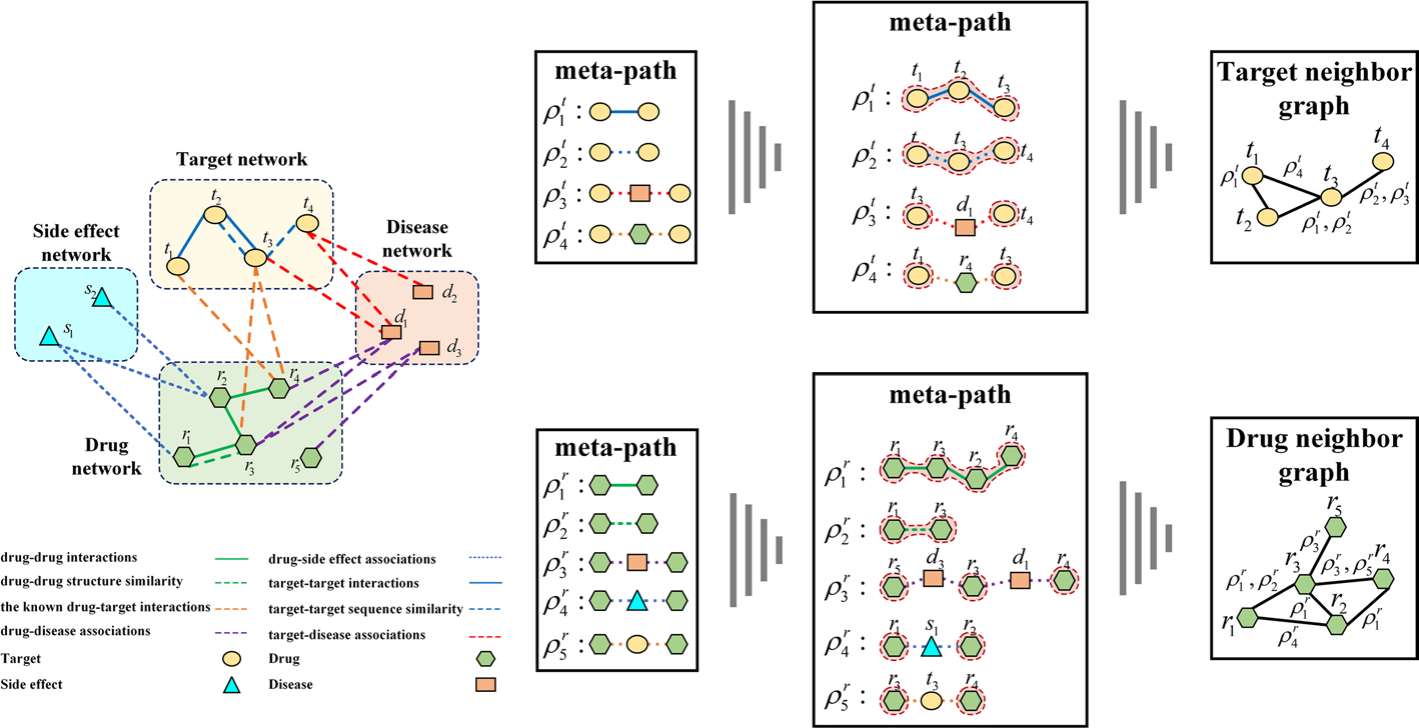
An illustration of a heterogeneous information network for DTIs

Taking the target as an example, we illustrate the construction method of the neighbor network using Fig. 2. Firstly, we extract subgraphs under four types of metapaths, and then combine these four subgraphs to obtain the neighbor graph of the protein. different metapaths can yield distinct sets of metapath-based neighbors for the same node. Additionally, in the heterogeneous information network, although there is no direct link between $t_{3}$ and $t_{1}$, they can still be connected through the metapath-based neighbors obtained from metapath. Hence, metapath-based neighbors offer valuable high-order connections between nodes, allowing for diverse semantic perspectives to be explored.

### Learning embedding with graph attention networks

In recent years, research has taken a dual approach: one stream has concentrated on exploring relationships between nodes [26, 27], while another has delved into the automatic acquisition of node-level latent feature representations (embeddings) that uphold the network topology. Since its inception by Veličković et al. [28], the GAT has garnered significant attention. GAT introduces an attention mechanism to compute the relationship weights between neighboring nodes and aggregates the features of all adjacent nodes into the central node. By assessing the consistency of topological structures between two nodes, GAT facilitates the extraction of local structural information from the graph.

To learn the embeddings of drugs and proteins, a two-layer GAT is employed. In the initial layer, specific meta-paths are utilized with GAT to learn drug embeddings ${\textbf{X}} ^{\rho _{i} }$. In the subsequent layer, an attention-based method is applied to integrate multiple drug embeddings, denoted as $\left\{ {\textbf{X}} ^{\rho _{1} },{\textbf{X}} ^{\rho _{2} },\ldots ,{\textbf{X}} ^{\rho _{s} } \right\}$, and generate the final drug embedding ${\textbf{X}}$. The same process is repeated to obtain the final embedding ${\textbf{Y}}$.

To elaborate further, let’s consider the meta-path $\rho$. In this particular context, $N^{\rho }(i)$ represents the collection of meta-path neighbors associated with a drug (or target) node $r_i$ (or $t_j$) within a heterogeneous information network. It’s worth noting that $j \in N^{\rho }(i)$ refers to a meta-path neighbor of *i*. The $l$-th layer’s embedding for *i* on meta-path $\rho$ is denoted as ${{\textbf{x}}}_i^{\rho } \in {\mathcal{R}}^{d_l}$, where $d_l$ represents the dimension of the embedding vector at the $l$-th layer. In this framework, the influence weight $w_{i,j}^{\rho }$ of *j* on *i* indicates the significance of node *j* in relation to node *i*. Notably, the weight $w_{i,j}^{\rho }$ is determined based on the embeddings of both *i* and *j*.

1 $$\begin{aligned} w_{i,j}^{\rho }=\sigma \left( {{\textbf{g}} } _{\rho }^{{\textsf{T}} } \cdot \left[ {\textbf{W}}^{\rho } {\textbf{x}}_{i}^{\rho }\left| \right| {\textbf{W}}^{\rho } {\textbf{x}}_{j}^{\rho } \right] \right) . \end{aligned}$$

In formula (1), $\sigma$ is activation function and the vector concatenation operation || are used in the process. Additionally, ${\textbf{g}}_{\rho }^{\top } \in {\mathcal{R}}^{2d_{l+1}}$ represents the influence weight vector of meta-path $\rho$, while ${\textbf{W}}^{\rho }\in {\mathcal{R}}^{d_{l+1}\times d_{l}}$ is a matrix of length $2d_{l+1}$. Additionally, ${\textbf{W}}$ represents the common linear transformation weight matrix. After obtaining the influence weight values for all meta-path neighbors, we calculate the attention coefficients $a_{i,j}^{\rho }$ by normalizing them with the softmax function.

2 $$\begin{aligned} a_{i,j}^\rho ={\text{softmax}}_{j\in N^\rho (i)}\Big (w_{i,j}^\rho \Big ) =\frac{\exp \Big (w_{i,j}^{\rho }\Big )}{\sum _{k\in N^{\rho }(i)}\exp \Big (w_{i,k}^{\rho }\Big )}. \end{aligned}$$

Then, the embedding of node *i* in the next ($l$+1)-layer can be calculated by aggregating the embeddings of its neighboring nodes in the $l$-layer, weighted by the attention coefficients. In other words, the weighted aggregation can be represented as follows:

3 $$\begin{aligned} {{\textbf{x}}}_i^\rho =\sigma \left( \sum _{j\in N^\rho (i)}\left( a_{i,j}^\rho \cdot {\textbf{W}}^\rho {{\textbf{x}}}_j^\rho \right) \right) . \end{aligned}$$

The *K*-head attention layer’s output can be obtained as follows, formula (4) where ${\textbf{x}}_j^{\rho }$ is the output by each head.

4 $$\begin{aligned} {\textbf{x}}_i^\rho =\Vert _{k=1\sim K}\sigma \left( \sum _{j\in N\rho (i)}\left( a_{i,j}^\rho \cdot {\textbf{W}}^\rho {\textbf{x}}_j^\rho \right) \right) . \end{aligned}$$

Formulas (1), (2), and (3) incorporate the trainable parameters ${\textbf{x}}_i^\rho$, ${\textbf{x}}_j^\rho$, and ${\textbf{g}}_{\rho }^{\top }$, which play a vital role in determining the values of $w_{i,j}^{\rho }$. These weights are essential for the model to assign higher aggregation weights to neighboring nodes that are more relevant to the DTIs prediction task. Consequently, the model can aggregate node embeddings based on these dynamic weights. The drug embeddings for all $l$-layers under the meta-path $\rho$ are represented as ${\textbf{G}}_{a}^{\rho }(l)\in {{\mathcal{R}}}^{n\times Kd_{l}}$. By employing graph attention, we can obtain ${\textbf{G}}_{a}^{\rho }(l+1)\in {\mathcal{R}}^{n\times Kd_{l+1}}$.

5 $$\begin{aligned} {\textbf{G}}_{a}^{\rho }(l+1)=\textrm{GAT}({\textbf{G}}_{a}^{\rho }(l)). \end{aligned}$$

The architecture of GAT for layer $l$ consists of multiple stacked graph attention layers.

### Learning embedding with residual graph convolution

Kipf introduced GCN to handle data with graph inputs [29]. Each convolutional layer in GCN is capable of processing information from the first-order neighborhood, thereby capturing vertex details from immediate neighbors. Through the stacking of multiple convolutional layers, GCN aggregates information from multiple-order neighborhoods to derive embedding representations for all vertices. The fundamental concept behind graph convolutional neural networks is to amalgamate information from a node’s own attributes and its neighboring nodes, with a focus on the connectivity between two nodes. This approach enables a more comprehensive understanding of each node, taking into account the structural information embedded in the data.

However, due to the issue of gradient vanishing in traditional GCN networks [30], we introduce residual graph convolutional networks in this section to learn node embeddings.

In this section, we provide an overview of the propagation rules for each layer of GCN as follows:

6 $$\begin{aligned} {\textbf{H}}(l+1)=\sigma \Big ({\textbf{D}}^{-\frac{1}{2}}{\textbf{A}} {\textbf{D}}^{-\frac{1}{2}}{\textbf{H}}(l){\textbf{W}}(l)\Big ). \end{aligned}$$

In the equation provided, ${\textbf{H}}(l+1)$ corresponds to the output of the ($l$+1)-th layer, while ${\textbf{W}}(l)$ denotes the trainable weight matrix of the $l$-th layer. We use $\sigma$ to represent the activation function. Within this expression, the matrix $\tilde{{\textbf{A}}}$ is computed by adding ${\textbf{A}}$ and ${\textbf{I}}_{N}$. Here, ${\textbf{I}}_{N}$ is an identity matrix with diagonal elements equal to 1 and non-diagonal elements equal to 0. Additionally, $\tilde{{\textbf{D}}}$ signifies the diagonal node degree matrix of $\tilde{{\textbf{A}}}$. To elaborate further on the node propagation process, consider the following:

7 $$\begin{aligned} h_i(l+1)=\sigma \left(\sum _{j\in Ne(i)}\frac{1}{c_{ij}}h_j(l){\textbf{W}}(l)\right). \end{aligned}$$

The potential representation of node *i* in the ($l$+1)-th layer is represented by $h_{i}(l+1)$. The set of neighbors of node *i* is denoted as $\text{Ne}($i), and $c_{i,j}$ stands for a normalization constant. Taking into account the cumulative impact from neighboring nodes, the update of features for each node can be articulated as follows:

8 $$\begin{aligned} h_i\leftarrow {\text{joint}}\left(\sum _jh_j\right). \end{aligned}$$

The initial layer of the GCN possesses a distinct nature. It accepts the adjacency matrix ${\textbf{A}}$ of the heterogeneous network as input, following symmetric processing. The feature matrix ${\textbf{X}}$ comprises both interaction and similarity features. The formulation for this initial layer is presented as follows:

9 $$\begin{aligned} {\textbf{H}}( l )=\sigma ({\textbf{A}}{\textbf{X}}{\textbf{W}}(0)). \end{aligned}$$

In the context of R-GCN, the learning process involves the estimation of the low-level mapping denoted as ${\textbf{H}}$, accomplished through the fitting of the residual mapping *F*. This is achieved by transforming ${\textbf{G}}_{c}^{\rho }(l)$ using the residual mapping *F* and adding it vertex-wise to obtain ${\textbf{G}}_{c}^{\rho }(l+1)$. The residual mapping $F({\textbf{G}}_{c}^{\rho }(l), {\textbf{W}}(l))$ takes the input graph and outputs the representation of the next layer’s residual graph, denoted as $G_{res}(l+1)$. The definition of ${\textbf{G}}_{c}^{\rho }(l+1)$ is as follows:

10 $$\begin{aligned} \begin{aligned} {\textbf{G}}_{c}^{\rho } (l+1)&={\textbf{H}}({\textbf{G}}_{c}^{\rho } (l),{\textbf{W}}(l)) \\&=F({\textbf{G}}_{c}^{\rho } (l),{\textbf{W}}(l))+{\textbf{G}}_{c}^{\rho } (l) \\&={\textbf{G}}_{c}^{res}(l+1)+{\textbf{G}}_{c}^{\rho } (l). \end{aligned} \end{aligned}$$

Here, $W_{l}$ denotes the collection of trainable parameters for the $l$-th layer.

To summarize, each module within the Relational R-GCN takes both the output from its preceding layer and the residual connection as input.

### Learning embedding with attention mechanism

By applying GAT transformation, we can derive the embedding matrix ${\textbf{X}}^{\rho _{i}}$ for all nodes in the heterogeneous information network given a metapath $\rho _{i}$. Hence, we obtain a set of node embeddings $\{{\textbf{X}}^{\rho _1},{\textbf{X}}^{\rho _2},\ldots ,{\textbf{X}}^{\rho _s}\}$ for a meta-path set $\{\rho _1,\rho _2,\ldots ,\rho _s\}$. Since different metapath semantics result in distinct metapath embeddings, integrating multiple metapath embeddings becomes necessary to obtain more comprehensive node embeddings. The metapath-level attention score $b^{\rho _i}$ for metapath $\rho _{i}$ is computed as per the following formula.

11 $$\begin{aligned} s^{\rho _i}=\dfrac{1}{n}\sum _{j\in V_d}{\textbf{q}}^{\top }\sigma \Big ({\textbf{W}}{\textbf{x}}_j^{\rho _i}+{\textbf{b}}\Big ). \end{aligned}$$

The weight matrix is denoted by ${\textbf{W}}$, the bias vector by ${\textbf{b}}$, the semantic-level attention vector by ${\textbf{q}}$, and the set of medicines is denoted by $V_{d}$ (the identical equation works for targets). It is important to acknowledge that, in order to conduct a valid comparison, all metapaths and specific semantic embeddings must possess the aforementioned parameters. The final attention values at the metapath level are derived by applying the softmax function to the attention scores mentioned above. This normalization process, as defined in Eq. (11), allows for the interpretation of the metapath embeddings’ respective contributions to the aggregated embedding.

12 $$\begin{aligned} a^{\rho _i}=\frac{\exp (s^{\rho _i})}{\sum _{j=1}^s\exp (s^{\rho _j})}. \end{aligned}$$

To obtain the final embedding ${\textbf{X}}$, we integrate the specific metapath embeddings using the learned attention values as coefficients. This integration is performed as follows:

13 $$\begin{aligned} {\textbf{X}}=\sum _{i=1}^s \left( \frac{a^{\rho _i} {\textbf{G}}_{a}^{\rho _i} +a^{\rho _i} {\textbf{G}}_{c}^{\rho _i} }{2} \right) . \end{aligned}$$

In summary, for a given set of metapaths $\{\rho _{1},\rho _{2},\ldots ,\rho _{s}\}$ in a heterogeneous information network for drugs, our learning approach starts with randomly initialized embeddings ${\textbf{G}}_{a}^{\rho _i}(0)$ and ${\textbf{G}}_{c}^{\rho _i} (0)$. For each metapath $\rho _{i}$, the embeddings undergo layer-wise transformations, and the final output is an attention-based aggregated embedding ${\textbf{X}}$.

### Predicting drug–target interactions with IMCHGAN

In this paper, the DTIs prediction is formulated as a neural network learning framework IMCHGAN as shown in Fig. 1. A matrix ${\textbf{T}}\in \{0,1\}^{m\times n}$ is created to reflect drug–target associations that are only partially seen. Each element of ${\textbf{T}}$ can take on a value of either 0 or 1. The matrix element ${\textbf{T}}(i,j)$ is equal to 1. The symbol indicates the presence of a recognized interaction between the variables $r_{i}$ and $t_{j}$. The matrix element ${\textbf{T}}(i,j)$ is equal to zero. The value of is currently undetermined or has not been detected in relation to the correlation between medicine *i* and target *j*. The task of predicting DTIs involves completing the absent entries within the partially observed matrix ${\textbf{T}}$. Matrix completion is a mathematical formulation for this task. However, it faces a limitation when it comes to using side information directly for DTI prediction. To tackle this problem, we introduce a method known as inductive matrix completion (IMC) [31]. In the context of utilizing IMC for DTI prediction, the associated prediction ratings are conceptualized as the inner product of drug and target features projected onto the latent space. IMC operates under the assumption that the association matrix is generated by applying feature vectors related to its row and column entities to the projection matrix ${\textbf{Z}}$. The primary objective is to recover ${\textbf{Z}}$ based on the observed values of ${\textbf{T}}$. To effectively learn parameters from a limited number of observed ratings, the latent space is constrained to be low-dimensional, implying that the parameter matrix is restricted to be low-rank. Consequently, the loss function for TTGCN can be formulated as follows:

14 $$\begin{aligned} &{\mathop {\textrm{min}}\limits _{{\textbf{Z}}_{1},{\textbf{Z}}_{2}}}{\frac{(1-\alpha ) }{2}}\Vert P_{\Omega }\big ({\textbf{T}}-{\textbf{X}}{\textbf{Z}}_{1}{\textbf{Z}}_{2}^{{\textsf{T}}} {\textbf{Y}}^{{\textsf{T}}}\big )\Vert _{F}^{2}\\&\quad \left. +\frac{\alpha *\mu }{2}\left\| P_{\bar{\Omega }}({\textbf{T}}-\textbf{XZ}_{1}{\textbf{Z}}_ {2}^{\top }{\textbf{Y}}^{\top })\right\| _{F}^{2}\right. . \end{aligned}$$

Here, $P_{\Omega }(\cdot )$ denotes the projection of the matrix onto the positive set $\varvec{\Omega }$. In our methodology, we have acquired feature matrices ${\textbf{X}}$ and ${\textbf{Y}}$ for targets and drugs, respectively. Our objective is to reconstruct the feature projection matrix ${\textbf{Z}}$ using the observed entries in the known drug–target association matrix ${\textbf{T}}$, along with the feature matrices ${\textbf{X}}$ and ${\textbf{Y}}$. To achieve this, we employ the factorization ${\textbf{Z}}={\textbf{Z}}_{1}{\textbf{Z}}_{2}^{\textrm{T}}$, where ${\textbf{Z}}_1$ and ${\textbf{Z}}_2$ are of rank $k\ll f_{\textrm{t}},f_{\textrm{r}}$ and have dimensions ${\mathcal{R}}^{f_{\text{t}}\times k}$ and ${\mathcal{R}}^{f_{\text{r}}\times k}$ respectively. To avoid generating degenerate results, the bias term is set to $\alpha \in (0,1)$. To address class imbalance, a small weight $\mu$ is assigned to the class 0, $\mu = \frac{\left\| \Omega \right\| }{\left\| \bar{\Omega } \right\| }$, $\bar{\Omega }$ is negative set.

## Experiments and discussions

### Evaluation metrics

To assess the algorithm’s performance, we conducted a 10-fold cross-validation as outlined [32]. In this process, the dataset containing a total of 708 drugs was randomly divided into 10 equally sized groups. Each group was designated as the test set, while the remaining nine groups served as the training dataset for model training. Following the prediction of interaction scores for all drug–protein pairs, the samples, namely drug–protein pairs, were arranged in descending order based on their scores. Higher rankings for the positive samples, which represent known DTIs, were indicative of superior model performance. Luo’s dataset encompasses 199,214 documented drug–disease associations, 5603 diseases, 1923 established DTIs, 1,512 proteins, and 708 drugs.

We assessed the prediction method’s performance using key metrics: the area under the receiver operating characteristic curve (AUROC) [33], the area under the precision–recall curve (AUPR) [34] and the matthews correlation coefficient (MCC). AUPR is often preferred when dealing with imbalanced data [35], making it a valuable evaluation measure. To further evaluate the method’s performance, precision–recall (PR) curves were also constructed.MCC is a metric used to evaluate the performance of classification models, which can avoid the limitations of relying solely on accuracy and provide more accurate model evaluation in situations with imbalanced samples.

To establish the statistical superiority of TTGCN, Wilcoxon tests [36] were conducted based on the AUROC and AUPR values for each drug in the dataset. Biologists typically identify potential DTIs by selecting those with higher interaction scores through wet lab experiments. Therefore, we collected the average recall at the top *k* (5%, 10%, 15%, 20%, 30%) to identify candidate samples for each method, showcasing their ability to uncover positive samples. Additionally, we used the average coverage as another metric to indicate how many steps the method requires to identify all known DTIs in the dataset. For each drug, the coverage value equals the number of samples queried when its recall reaches 1.

For simplicity, we’ll use the abbreviations TP (true positives), FP (false positives), TN (true negatives), and FN (false negatives). The formulas for the area under the ROC curve (AUC), true positive rate (TPR), false positive rate (FPR), precision, recall are as follows, and matthews correlation coefficient (MCC):

15 $$\begin{aligned} TPR= {} \frac{TP}{TP+FN}, \end{aligned}$$

16 $$\begin{aligned} FPR= {} \frac{FP}{FP+TN}, \end{aligned}$$

17 $$\begin{aligned} Precision= {} \frac{TP}{TP+FP}, \end{aligned}$$

18 $$\begin{aligned} Recall= {} \frac{TP}{TP+FN}. \end{aligned}$$

19 $$\begin{aligned} MCC= {} \frac{TP\times TN- FP\times FN }{\sqrt{\left( TP+ FP \right) \left( TP+ FN \right) \left( TN+ FP \right) \left( TN+ FN \right) } }. \end{aligned}$$

### Compared methods and parameters setting

TTGCN was compared against several state-of-the-art DTI prediction methods, including GRMF [37], DTINet [15], MolTrans [17], and NGDTP [38].

The hyperparameters for each method under comparison were selected from the suggested range specified in the respective literature. In accordance with the outcomes of our experiments, we set the hyperparameters as follows:

- For GRMF, we set $\lambda _{d}=\lambda _{p}=0.1$ and $\lambda _{l}=0.2$.
- In DTINet, the restart probability for random walk was set to $r=0.8$, and $k_{1}=100$ and $k_{1}=400$ were used.
- NGDTP was configured with $f_{r}=280$ and $f_{r}=210$ in the matrix decomposition step, and $a_{1}=a_{2}=a_{3}=0.1$. I
- In the GBDT model, we set $num_{leaves}=80$ and a learning rate of 0.02.
- For MolTrans, we used a Batch Size of 16, Learning Rate of 0.0001, Epoch of 30, and Dropout of 0.1.

TTGCN was trained and optimized using PyTorch on a GPU device (Nvidia GeForce GTX 3070). To identify the best hyperparameters, we experimented with various choices:

- Activation functions were tested, including Sigmoid, ReLU, Leaky ReLU, and Tanh.
- Batch sizes were considered from the range $\{16, 32, 64, 128, 256\}$.
- Learning rates were chosen from $\{10^{-2}, 10^{-3}, 10^{-4}$, $10^{-5}\}$.
- Dropouts were chosen from $\{0.2,0.3, 0.4, 0.5\}$.

Based on the AUROCs and AUPRs obtained with different parameter configurations, we ultimately settled on Tanh as the activation function. Additionally, we selected Batch Size of 64, Learning Rate of 0.001, Epoch of 1000, and Dropout of 0.4 for the final model configuration after comparing the experimental results.

### Experimental comparison

**Fig. 3:**
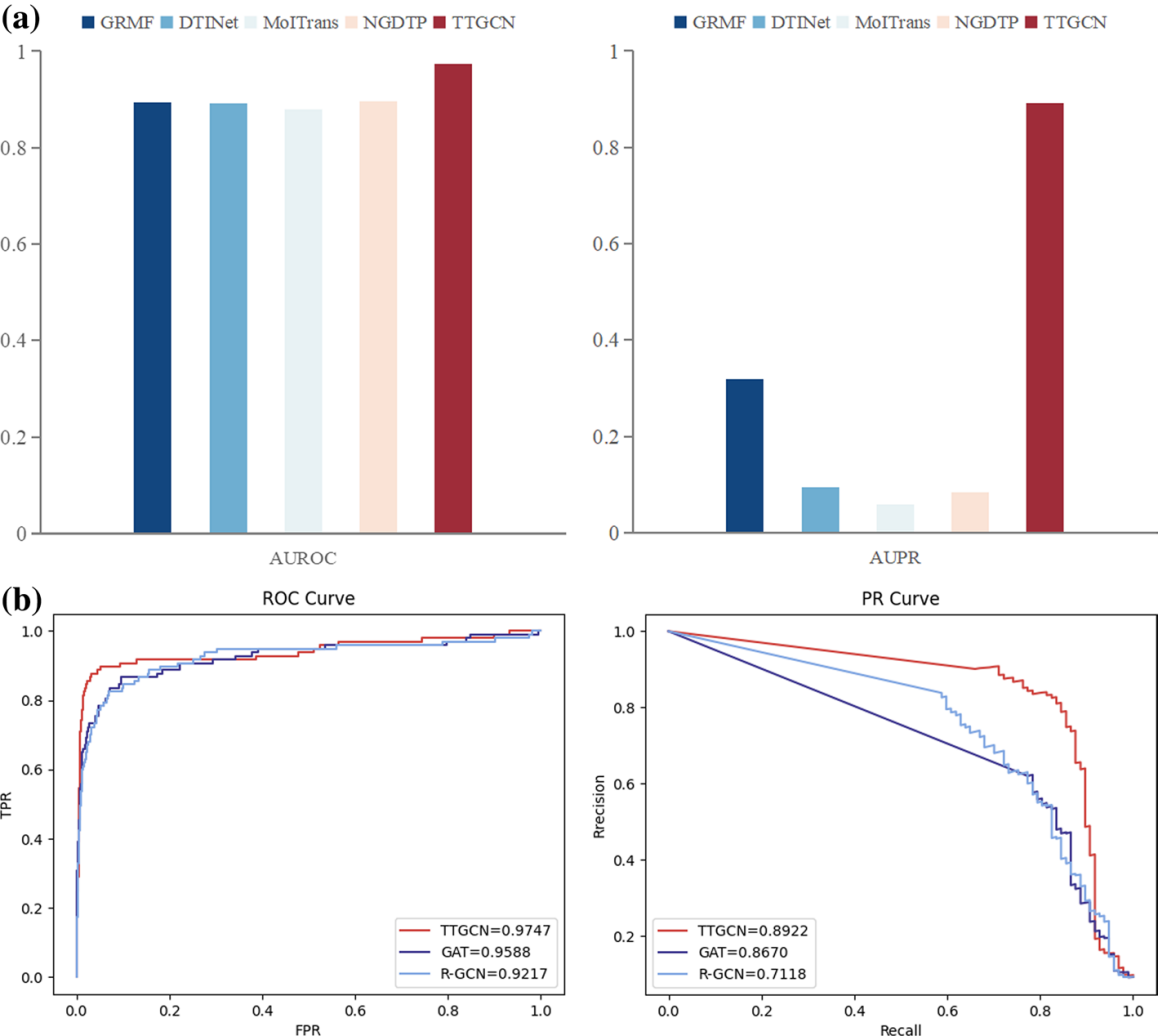
**a** Evaluation of ROC and PR for TTGCN in comparison to four state-of-the-art DTI prediction methods. **b** Assessment of ROC curves and PR curves in the context of the ablation experiment

**Fig. 4:**
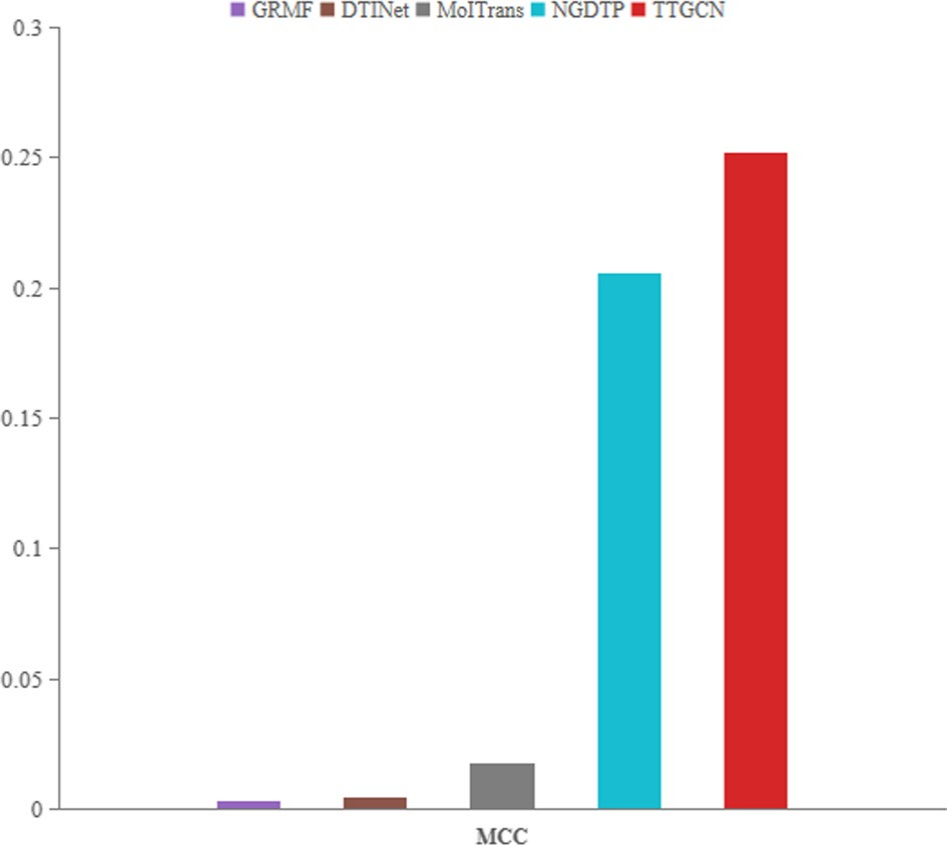
Evaluation of MCC for TTGCN in comparison to four state-of-the-art DTI prediction methods

**Fig. 5:**
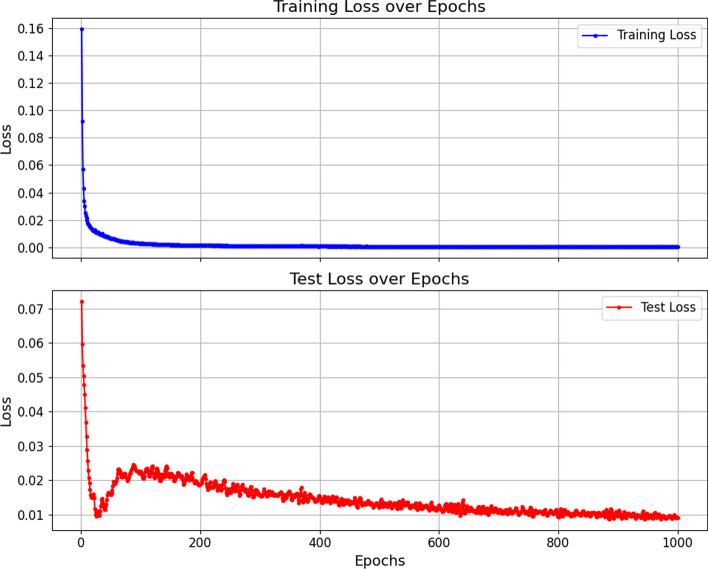
Loss function graph

The ROC and PR bar charts for each method are illustrated in Fig. 3a. In our dataset, TTGCN utilizes GAT to preserve connectivity between nodes, while R-GCN ensure connectivity between two nodes in both the original feature space and the embedded space. This ensures a consistent topological structure between the original feature space and the embedding space, contributing to TTGCN’s exceptional performance with an AUROC of 97.5% and AUPR of 89.2%. TTGCN outperforms the second-ranking method, IMCHGAN, by 1.59% and 2.52% in AUROC and AUPR, and surpasses DTINet by 8.11% and 79.71%, respectively. MolTrans exhibits AUROC and AUPR of 87.9% and 5.9%, respectively, which are 9.6% and 83.3% lower than TTGCN.The bar chart in Fig. 4 shows the MCC for each method. TTGCN performs the best in terms of MCC and displays the ability to handle imbalanced datasets. Additionally, to evaluate the effectiveness of our model’s training process, we plotted the convergence curve of the loss, as shown in Fig. 5.

In cases where there is a severe class imbalance in the dataset (with a positive-to-negative sample ratio of 1:555), MolTrans’s performance may deteriorate. This is because the model can only be trained using an equal number of negative samples as positive samples, resulting in the exclusion of a substantial number of negative samples that could potentially contain valuable information. Despite NGDTP being based on a shallow model and achieving 7.8% lower AUROC and 80.9% lower AUPR compared to TTGCN, its utilization of ensemble learning allows it to fully leverage the negative samples. Notably, NGDTP outperforms MolTrans by 2.4% in AUPR. In comparison to GRMF, TTGCN demonstrates substantial improvements with an 8.1% higher AUROC and a 57.4% higher AUPR. The inferior performance of GRMF is likely attributed to both the limited learning capacity of the shallow model and the disregard for the inherent attributes of the drug and protein nodes.

To demonstrate the importance of both connectivity between two nodes and having consistent topological structures, we conducted an ablation experiment. The experimental results (Fig. 3b) showed that compared to not using the GAT model, the adopted AUROC and AUPR improved by 5.3$\%$ and 18.0$\%$ respectively. Compared to not using the graph convolutional neural network model, the adopted AUC and AUPR improved by 1.6$\%$ and 2.5$\%$ respectively. This indicates that considering the connectivity between two nodes and having consistent topological structures can effectively improve the accuracy of the model. Therefore, exploring the connectivity between two nodes and whether they have consistent topological structures is necessary.

**Table 1:** The proportions of drugs with AUROC or AUPR values exceeding the threshold $\delta$

	AUROCs			AUPRs		
	$\delta =0.9$	$\delta =0.8$	$\delta =0.7$	$\delta =0.8$	$\delta =0.5$	$\delta =0.3$
GRMF($\%$)	**80**.**8**	83.7	86.8	**43**.**6**	52.7	53.0
DTINet($\%$)	77.5	82.4	88.5	17.0	23.6	26.9
MolTrans($\%$)	73.9	85.0	88.5	17.7	22.1	26.5
NGDTP($\%$)	59.8	82.2	**96**.**9**	1.0	5.6	17.6
TTGCN($\%$)	62.5	**86**.**9**	95.04	30.8	**64**.**8**	**83**.**4**

Bold is the threshold

**Table 2:** The statistical significance of the improvement of TTGCN over other methods in terms of AUC and AUPR (Wilcoxon test)

	DTINet	GRMF	NGDTP	MolTrans
P-value (AUROCs)	2.72e–29	4.92e–24	3.73e–30	1.67e–39
P-value (AUPRs)	2.69e–11	3.63e–3	2.02e–3	1.08e–20

We’ve summarized the impact of each prediction method on individual drugs, and Table 1 displays the percentage of drugs with AUROC or AUPR surpassing the threshold $\delta$. To assess the statistical significance of TTGCN’s performance in terms of AUROC and AUPR, we conducted Wilcoxon tests. Specifically, we calculated the AUROC and AUPR for drugs in the dataset under various DTI prediction methods, based on predicted scores for each target. Subsequently, we computed P-values using Wilcoxon tests to compare TTGCN with each of the other methods, taking into account AUROC and AUPR. The results, presented in Table 2, demonstrate that TTGCN significantly outperforms other methods in both AUROC and AUPR when the P-value threshold is set at 0.05.

**Fig. 6:**
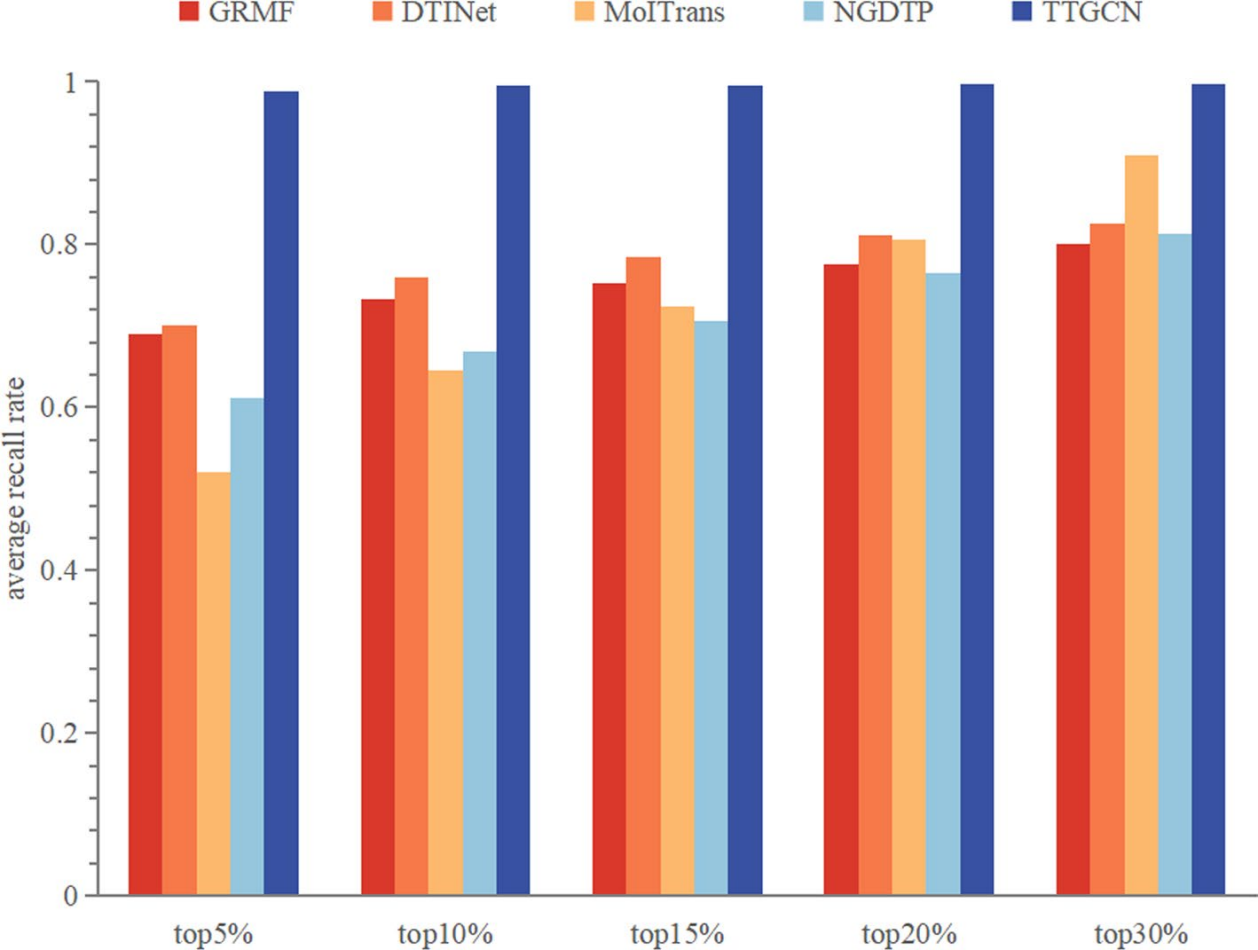
The mean recall rates for various top-*k* thresholds

**Fig. 7:**
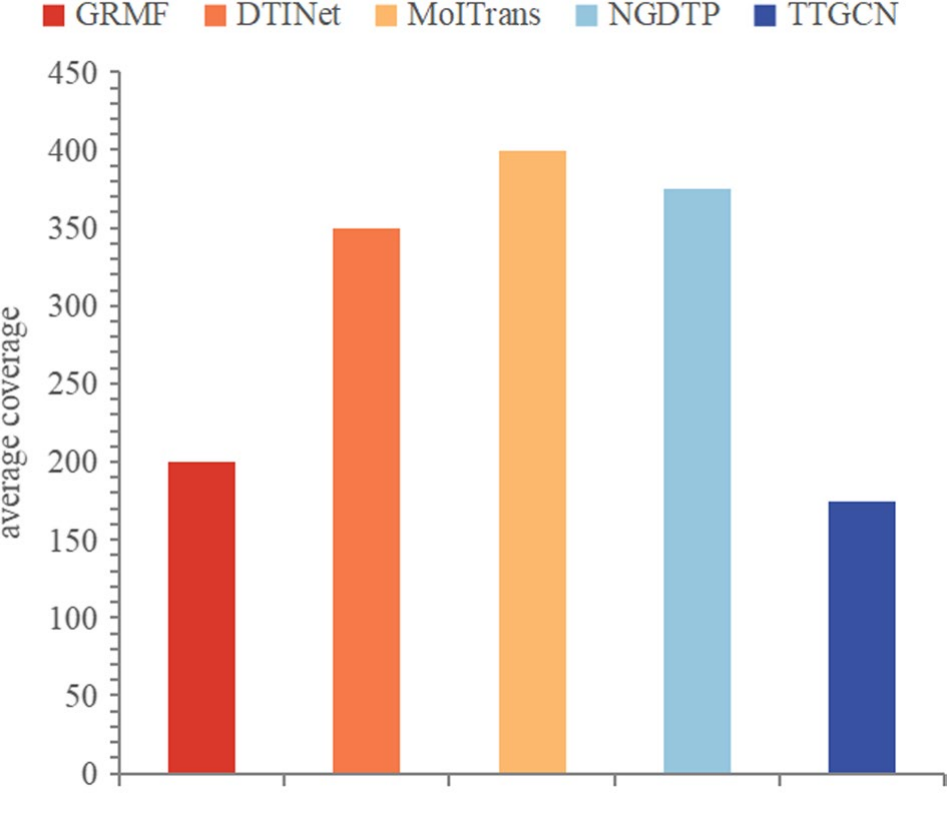
The mean coverage for each approach

The recall rate, particularly at the upper portion of the predicted results, serves as an indicator of the model’s capacity to uncover DTIs. Figure 6 illustrates the average recall rates of the top *k* candidates (where *k* takes values of 5, 10, 15, 20, and 30) for each method. As the recall rate increases, so does the number of genuine DTIs successfully identified by the prediction method. Consequently, TTGCN exhibits superior DTI discovery capabilities compared to other methods, boasting the highest recall rate at each cutoff, as depicted in Fig. 6. Figure 7 presents the average coverage for each method, with lower coverage indicating a swifter discovery of all latent DTIs by the model. Thus, TTGCN proves to be more potent and efficient than alternative methods in the detection of potential DTIs.

### TTGCN predicts novel DTIs

To evaluate its effectiveness, TTGCN was trained using the complete set of known DTIs in the dataset and subsequently employed to predict target proteins for all drugs.

**Table 3:** Candidate drug–target pairs

Rank	Drug ID	Protein ID	Supported evidence	Rank	Drug ID	Protein ID	Supported evidence
1	DB00396	P10275	UniProt	11	DB00321	P28221	UniProt
2	DB00201	P27815	DrugCentral	12	DB00321	P35368	Drugbank
3	DB00396	P04150	UniProt	13	DB00696	P08908	UniProt
4	DB00321	P28335	UniProt	14	DB00321	Q9H3N8	UniProt
5	DB00321	P35372	DrugCentral	15	DB00839	Q09428	DrugCentral
6	DB00929	P34995	DrugCentral	16	DB00408	P28222	UniProt
7	DB00321	P28222	UniProt	17	DB00242	P27707	UniProt
8	DB00418	Q13002	UniProt	18	DB00988	P31645	UniProt
9	DB00201	Q08499	DrugCentral	19	DB00800	P35348	Drugbank
10	DB00321	P50406	UniProt	20	DB00986	P35348	UniProt

To validate these predictions, we searched for supporting evidence in three databases: DrugBank, DrugCentral, and UniProt. These databases include information obtained from the planning, experimentation, and publication of DTIs. Table 3 displays both the predicted and validated results, highlighting the robust predictive capabilities of TTGCN.

## Conclusion

In this study, we introduce an approach that leverages multiple drug features and incorporates GAT and R-GCN to predict potential DTIs. TTGCN employs graph attention and residual graph convolutional neural networks to learn latent feature representations in the biological information network. It pays more attention to the connectivity between two nodes and whether the topological structure is consistent between the original feature space and the embedding space. Moreover, compared to single graph attention and single residual graph convolutional neural network models, TTGCN excels in uncovering previously unknown connections between drugs and target proteins.

The experimental findings unequivocally establish TTGCN’s superior performance over numerous state-of-the-art DTI prediction methods. Moreover, the predictions made by TTGCN have been validated to include a substantial number of authentic DTIs. These outcomes underscore TTGCN as a compelling option for biologists seeking to identify dependable candidate DTIs for subsequent wet laboratory experiments. Currently, our model is used for predictions of drugs and proteins. In the future, we will explore relationships among various biological entities.

## Data Availability

The datasets generated during the current study are acquired from multiple scholarly articles, i.e. [22–24].
